# A Crohn’s Disease-associated IL2RA Enhancer Variant Determines the Balance of T Cell Immunity by Regulating Responsiveness to IL-2 Signalling

**DOI:** 10.1093/ecco-jcc/jjab103

**Published:** 2021-06-12

**Authors:** Rimma Goldberg, Jennie N Clough, Luke B Roberts, Jenifer Sanchez, Shahram Kordasti, Nedyalko Petrov, Arnulf Hertweck, Anna Lorenc, Ian Jackson, Scott Tasker, Anna Appios, Omer Omer, Miles Parkes, Natalie Prescott, Richard G Jenner, Peter M Irving, Graham M Lord

**Affiliations:** 1 School of Immunology and Microbial Sciences, King’s College London, London, UK; 2 School of Clinical Sciences, Monash University, Melbourne, VIC, Australia; 3 Department of Gastroenterology, Monash Health, Melbourne, VIC, Australia; 4 IBD Unit, Gastroenterology Department, Guy’s and St Thomas’ NHS Trust, London, UK; 5 National Institute for Health Research Biomedical Research Centre, Guy’s and St Thomas’ NHS Trust and King’s College London, London, UK; 6 CRUK-KHP Cancer Centre, School of Cancer and Pharmaceutical Sciences, King’s College London, London, UK; 7 UCL Cancer Institute, University College London, London, UK; 8 Department of Medicine, Addenbrooke’s Hospital, University of Cambridge, Cambridge, UK; 9 Medical and Molecular Genetics, Kings College London , London, UK; 10 Faculty of Biology, Medicine and Health, University of Manchester, UK

**Keywords:** Crohn’s disease, T cells, TREGs, CD25, IL-2, basiliximab

## Abstract

**Background and Aims:**

Differential responsiveness to interleukin [IL]-2 between effector CD4^+^ T cells [T_eff_] and regulatory T cells [T_reg_] is a fundamental mechanism of immunoregulation. The single nucleotide polymorphism [SNP] rs61839660, located within *IL2RA* [CD25], has been associated with the development of Crohn’s disease [CD]. We sought to identify the T cell immune phenotype of IBD patients who carry this SNP.

**Methods:**

T_eff_ and T_reg_ were isolated from individuals homozygous [TT], heterozygous [CT], or wild-type [CC] for the minor allele at rs61839660, and used for phenotyping [flow cytometry, Cytometry Time Of Flight] functional assays or T cell receptor [TCR] sequencing. Phosphorylation of signal transducer and activator of transcription 5 [STAT5] was assessed in response to IL-2, IL-7, and in the presence of basiliximab, a monoclonal antibody directed against CD25. T_eff_ pro-inflammatory cytokine expression levels were assessed by reverse transcription quantitative polymerase chain reaction after IL-2 and/or TCR stimulation.

**Results:**

Presence of the minor T allele enhances CD25 expression, leading to increased STAT5 phosphorylation and pro-inflammatory cytokine transcript expression by T_eff_ in response to IL-2 stimulation *in vitro*. T_eff_ from TT individuals demonstrate a more activated gut homing phenotype. TCR sequencing analysis suggests that TT patients may have a reduced clonal capacity to mount an optimal regulatory T cell response.

**Conclusions:**

rs61839660 regulates the responsiveness of T cells to IL-2 signalling by modulating CD25 expression. As low-dose IL-2 is being trialled as a selective T_reg_ modulator in CD, these findings highlight the potential for adverse effects in patients with this genotype.

## Graphical Abstract

**Graphical Abstract:**
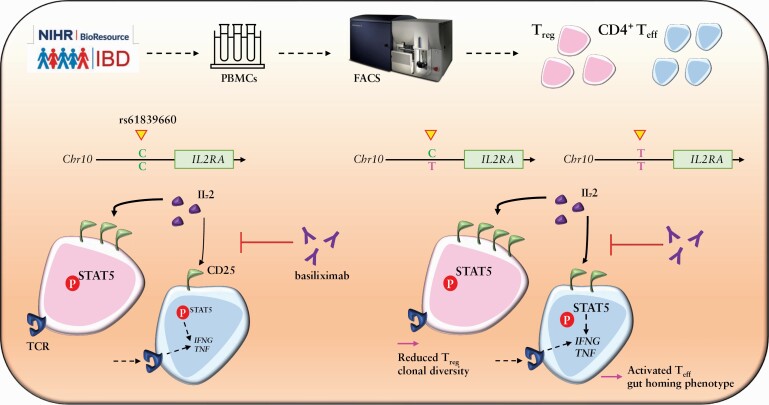

## 1. Introduction

Crohn’s disease [CD] is a complex immune-mediated disorder with polygenic inheritance, in which inappropriate activation of the intestinal immune system in a genetically susceptible individual triggers chronic inflammation of the gastrointestinal tract. Despite the identification of multiple associated genetic polymorphisms,^1^ few have been mapped to a mechanistic pathway and none have yet yielded a tractable therapeutic approach for CD patients. Recent years have seen an increasing array of treatment options with the advent of biologic therapy, including anti-tumour necrosis factor [TNF]-α and anti-integrin agents. However, rates of surgery and hospitalisation for CD patients have not significantly changed in the biologic era.^2^ Therefore, there is a pressing need to explore novel therapeutic pathways. The value of a personalised approach to treatment is increasingly recognised, with the aim of targeting a treatment at the dominant aberrant pathway in a given patient to maximise efficacy and minimise side effects.^3^ The IL-2 pathway has been identified as potentially therapeutically tractable in numerous autoimmune conditions, including inflammatory bowel disease [IBD], with recruitment to a trial of low-dose IL-2 in CD under way [ClinicalTrials.gov NCT01988506].

Regulatory [T_reg_] and effector [T_eff_] CD4^+^ T cells are important mediators of the immune response in the gut of IBD patients, with defects in T_reg_ number and suppressive function noted in the lamina propria and peripheral blood of patients with active IBD.^4,5^ Additionally, many identified IBD genetic risk loci map to immune cell enhancer regions, with particular enrichment in CD4^+^ T cell enhancers.^6^ T_reg_ are characterised by high constitutive expression of the IL-2 receptor alpha chain [*IL2RA,* CD25], a component of the high-affinity IL-2 receptor heterotrimer [IL2Rα/β/γ]. The lineage-defining transcription factor Forkhead box P-3 [FOXP3] is essential for their suppressive phenotype and stability.^7,8^ An additional defining feature of T_reg_ is their low expression, or absence, of the IL-7 receptor alpha chain [CD127].^9^ In homeostatic conditions, IL-2 is mainly produced by activated CD4^+^ T cells in secondary lymphoid organs and is consumed at the same site by cells that express CD25, of which the majority are T_reg._^10^ By contrast, CD4^+^ T_eff_ express CD25 only after activation, and at relatively lower levels compared with T_reg_, suggesting that T_reg_ can preferentially respond to low concentrations of environmental IL-2. This differential responsiveness to IL-2 signalling is an important mechanism of immunoregulation.

Over 200 risk loci for IBD have been identified through genome-wide association studies [GWAS], with many in key regulatory pathways.^1,11^ The single nucleotide polymorphism [SNP] rs61839660 denotes a cytosine [C] to thymidine [T] base change on chromosome 10, located within a putative intronic enhancer region [intron 7] of *IL2RA*. rs61839660 was initially identified through GWAS, and is one of a minority of SNPs to be confidently resolved through fine-mapping as a variant highly associated with CD; 9.4% of CD patients carry the risk allele,^12^ which equates to almost 18 000 CD patients in the UK.^13^ rs61839660 is also associated with the development of other autoimmune conditions including ankylosing spondylitis, psoriasis, and primary sclerosing cholangitis,^6,14^ demonstrating its importance for core immunoregulatory processes.

Previous experimental work has generated conflicting results on the effect of rs61839660 on CD25 expression in CD4^+^ T cells. A study in healthy human volunteers found that carriage of the rs61839660 minor allele was associated with increased levels of *IL2RA* messenger mRNA [mRNA] and increased CD25 surface expression on CD4^+^ memory T cells.^14^ However, contemporaneous studies in which rs61839660 was delivered into murine CD4^+^ T cells via CRISPR-based methods, in addition to studies of healthy human carriers, reported that the minor variant of rs61839660 was associated with reduced CD25 transcript levels in CD4^+^ T cells stimulated with anti-CD3/CD28.^15^ This group hypothesised, therefore, that rs61839660 impairs the function of the intronic enhancer that regulates CD25 expression in response to T cell receptor [TCR] stimulation of T_eff._ We sought to define the immune phenotype associated with rs61839660 in CD patients, with the aim of understanding potential therapeutic targets in this patient cohort.

## 2. Materials and Methods

**Figure 3.**
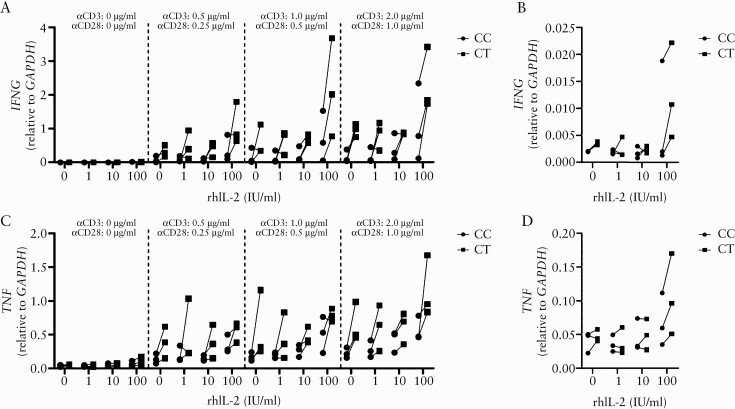
The rs61839660 minor allele enhances transcription of pro-inflammatory cytokines following exogenous IL-2 and acute TCR stimulation of CD4^+^ effector T cells. CD4^+^ effector T cells [T_eff_] from heterozygous [CT] and major allele homozygous [CC] patients were treated with different doses of recombinant human IL-2 for 24 h before being stimulated for 6 h [still in the presence of IL-2] with different concentrations of plate-bound **α**CD3/CD28. [a] Relative expression of *IFNG* in T_eff_ from the two genotypes investigated after the specified stimulations. [b] Relative expression of *IFNG* after 24 h of incubation with different concentrations of IL-2 in absence of TCR stimulation. [c] Relative expression of *TNF* in T_eff_ from the two genotypes investigated after the specified stimulations. [d] Relative expression of *TNF* after 24 h of stimulation with different concentrations of IL-2 in absence of TCR stimulation; *n* = 3 individual Crohn’s disease patients per genotype. Results shown are the summary from three independent experiments using *n* = 1 of each genotype per experimental run. Connected points are comparative samples from the same experimental conditions, run in the same experiment. Relative expression values are normalised to *GAPDH*. TCR, T cell receptor.

**Figure 4.**
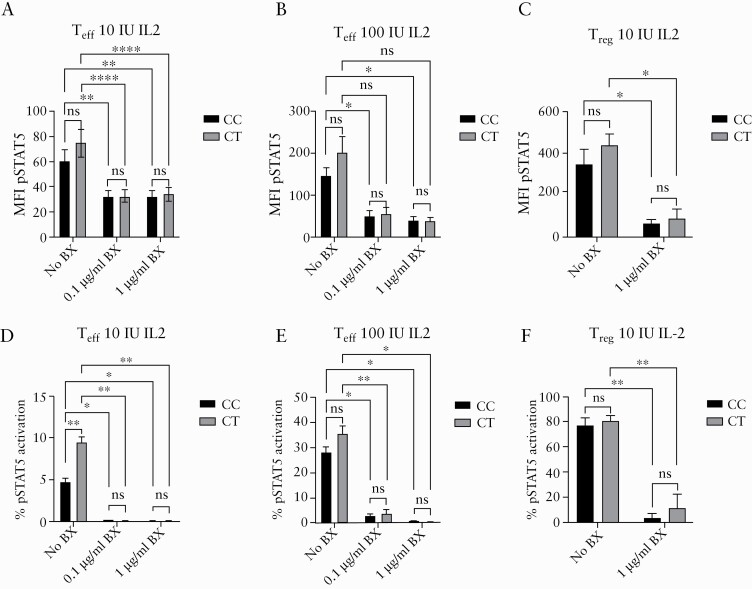
Basiliximab is effective at blocking phosphorylation of STAT5 in response to IL-2 signalling in rs61839660 heterozygote CD4^+^ T cells. Basiliximab was added to *in vitro* cultures of CD4^+^ effector T cells [T_eff_] and regulatory T cells [T_reg_] from heterozygous [CT] and major allele homozygous [CC] Crohn’s disease [CD] patients [n = 3 of each genotype], at the specified concentrations. After 6 h, cells were stimulated with 10 or 100 IU/ml of recombinant human IL-2 for 15 min. [a] Basiliximab was effective in reducing signal transducer and activator of transcription 5 [STAT5] phosphorylation [pSTAT5], quantified as a decrease in the mean fluorescence intensity [MFI] of pSTAT5 flow cytometry staining in T_eff_ stimulated with [a] 10 IU/ml IL-2 or [b] with 100 IU/ml of IL-2. There were no significant differences in basiliximab response between the genotype groups. [c] Basiliximab reduced the MFI for pSTAT5 staining in T_reg_ stimulated with 10 IU of IL-2 in CT and CC subjects. [d] Proportion [%] of pSTAT5 stained T_eff_ activated with 10 IU/ml and [e] 100 IU/ml IL-2 following basiliximab treatment. [f] Proportion [%] of pSTAT5 stained T_reg_ after stimulation with 10 IU/ml IL-2 following basiliximab treatment. Mean ± SEM plotted. Statistical analyses performed using two-way ANOVA, Tukey’s multiple comparisons test for comparisons involving more than two groups, and paired t tests for comparisons involving two treatments within the same genotype. *p <0.05, **p <0.01, ****p <0.0001. BX, basiliximab; Ns, not significant; SEM, standard error of the mean; ANOVA, analysis of variance.

Due to the Covid-19 pandemic and the closure of many patient-facing services in the UK, we were unable to source TT homozygote patient samples for certain experiments included in our study. Specifically, at certain times during which our work was carried out, the UK-wide IBD BioResource, upon which this study is based, was unable to continue recruitment based on UK Government legislation. Therefore, we were obliged to focus certain experiments on patients who are heterozygous for the minor risk allele at rs61839660, which represents nearly 10% of all patients with Crohn’s disease worldwide. The data from these experiments are presented in Figures 3 and 4 of this study.

Ethics approval for human blood and tissue collection was obtained from NRES Committee – London Riverside [REC reference: 15/LO/0151] and Guy’s and St Thomas’ NHS Trust R&D [R&D REF: RJ115/N122].

Permission was obtained from the NIHR IBD Bioresource to conduct a study of T_reg_/T_eff_ phenotype and function in patients and healthy controls [HC] carrying the CD-associated single nucleotide polymorphism [SNP] rs61839660. Individuals homozygous [TT], heterozygous [CT], or wild-type [CC] voluntarily provided a whole blood sample. Baseline clinical information collected included age, gender, disease duration and location, and treatment history.

### 2.1. Peripheral blood mononuclear cell isolation

Peripheral blood was layered over Lymphocyte Separation Medium [LSM 1077; PAA] followed by centrifugation at 2000 rpm for 30 min at 20°C with slow acceleration and no brake. The interface was aspirated and washed with sterile PBS at 1800 rpm for 10 min at 4°C with normal acceleration and deceleration. Cell count and viability were confirmed with trypan blue staining [Sigma-Aldrich].

### 2.2. Cell sorting

A proportion of peripheral blood mononuclear cells [PBMCs] were stained with Human Regulatory T Cell Sorting Kit [BD Biosciences, San Diego, CA, USA], according to the manufacturer’s instructions. Enriched CD4^+^ cells were labelled with mouse anti-human CD4-PerCpCy5.5 [clone L200], anti-CD25^-^phyroerythrin [PE] [clone 2A3], anti-CD127-AlexaFluor 647 [clone 40131.111], and CD45RA-FITC [clone HI100]. Cells were sorted into CD4^+^CD25^hi^CD127^lo^ T_reg_ and CD25^lo^CD127^hi^ T_eff_ on a FACSAria [BD Biosciences]. A FOXP3 stain was performed to ensure that the flow sort was selecting a true T_reg_ population [gating strategy, Supplementary Figure 1, available as Supplementary data at *ECCO-JCC* online].

### 2.3. Functional pSTAT5 assays

To ascertain the functional relevance of rs61839660, pSTAT5 assays were performed on sorted T_reg_ and T_eff_ of subjects of each genotype. PBMCs were defrosted the day before the experiment and rested overnight in XVIVO-15 [Lonza], at 37^°^C, 5% CO_2_. On the day of the experiment, cells were counted and 4 x 10^6^ were kept aside for cytometry time of flight [CyTOF] staining. The remainder were washed and stained for flow sorting as described above. T_reg_ and T_eff_ from each sample were sorted into XVIVO-15 and taken forward into the pSTAT5 assay.

In the first experimental phase, the assay was optimised on T_reg_ and T_eff_ sorted from a healthy control [HC] [blood bank cone]. Three concentrations of recombinant human [rh]IL-2 were used [1, 10, or 100 IU/ml per 1 x 10^5^ cells] and each concentration was assessed at three time points [15, 20, and 30 min]. An unstimulated sample was run in parallel. 10 IU/ml IL-2 for 15 min yielded a robust pSTAT5 response, but not the maximal response. This was selected as the most appropriate initial concentration to assess pSTAT5 response without saturating the pathway, to permit discrimination of differences in T_reg_ and T_eff_ responses.

As a control, 1 x 10^5^ sorted T_reg_ and T_eff_ from each subject were stimulated with recombinant human IL-7 across a logarithmically increasing dose range of concentrations [0, 1, 10, 100 IU/ml] under the same conditions. We hypothesised that, as IL-7 signals through CD127 and not through CD25, there should be no difference in the pSTAT5 response seen across genotypes.

For STAT5 phosphorylation assays, the BD Phosphoflow Protocol for human PBMCs was followed. Cells were washed, re-suspended in X-VIVO15, and rested for 1 h at 37^°^C, 5% CO_2_, before stimulation with IL-2 for 15 min in a 37^°^C water bath. Cells were fixed with warmed Cytofix buffer and incubated at 37^°^C for 10 min, then centrifuged at 600 x *g* for 5 min, supernatant discarded, and re-suspended in 100 μl Perm Buffer III. Cells were transferred to a 96-well v bottom plate and incubated on ice for 30 min, before washing and staining with 1 µl pSTAT5 AF488 (BD, clone 47/Stat5[pY694]) in 10 μl Stain Buffer, and incubated at room temperature for 60 min. Finally, cells were washed and re-suspended in 100 μl of PBS for acquisition on a Fortessa [BD]. Standardised acquisition settings were put in place and used in all subsequent experiments. Gates for the pSTAT5-positive population were set against an fluorescence minus one [FMO] control and the unstimulated sample.

### 2.4. **Assessment of pro-inflammatory cytokine transcript expression by CD4+ T**_**eff**_**in response to IL-2 and acute T cell receptor stimulation.**

T_eff_ were isolated by fluorescence-activated cell sorting [FACS] as described. Cells were cultured at 37^o^C/5% CO_2_ at a density of 4 x 10^5^–1 x 10^6^ cells / ml in U bottom 96-well cell culture plates [Corning Costar] for 24 h with 0, 1, 10, or 100 IU/ml rhIL-2 in 200 µl RPMI [Gibco] + 10% fetal calf serum [FCS] + 2 mM L-glutamine + 10 mM HEPES + 1 x non-essential amino acids + 1 mM sodium pyruvate + 50 U/ml penicillin + 50 µg/ml streptomycin + 50µM β-mercaptoethanol. Following this, cultures were transferred to wells of a 96-well flat-bottom plate, pre-coated with the indicated concentrations of αCD3 [clone OKT3] and αCD28 [clone CD28.2], and briefly centrifuged to quickly settle the cell layer. Following 6 h of culture, samples were removed from the activation plates, washed in ice-cold 1 x PBS [without Ca^2+^/Mg^2+^], re-suspended in 700 µl Qiazol [Qiagen], incubated for 30 min at room temperature, vortexed thoroughly for 15 s and frozen at -70^o^C for a minimum of 24 h before further use.

### 2.5. Reverse-transcription quantitative polymerase chain reaction

T_eff_ frozen in Qiazol were defrosted and RNA was isolated and purified via chloroform extraction using the miRNeasy Micro Kit, following the manufacturer’s protocol for isolation of total RNA from small samples [Qiagen]. RNA concentration was quantified using a Nanodrop spectrophotometer [Thermofisher Scientific]; 100 ng of total RNA per sample was reverse-transcribed using the RevertAid First Strand cDNA Synthesis Kit and random hexamer primers according to the manufacturer’s protocol. Quantitative polymerase chain reaction [qPCR] was performed using a ViiA 7 real-time PCR thermocycler [Applied Biosystems] in a final volume of 10 µl, using the 2 x Maxima probe/ROX qPCR Master Mix [Thermofisher Scientific] and the cDNA equivalent of 5 ng total RNA per sample. Detection of transcripts for pro-inflammatory cytokines was carried out using FAM-labelled 20 x TaqMan probe assays [Thermofisher Scientific] for *IFNG* [assay ID: HS00989291_m1] and *TNF* [assay ID: HS00174128_m1] and relative expression of each gene was calculated using the comparative cycle threshold [Ct] method [2^-ΔCt^] using *GAPDH* [assay ID: HS99999905_m1] as the endogenous reference control. Reactions were run in duplicates and the average Ct value for each reaction was used for analysis.

### 2.6. Basiliximab suppression assays

Optimisation work was performed to identify basiliximab conditions that would permit discrimination of CD25 blockade and subsequent pSTAT5 suppression between samples [HC blood bank cone, two replicates performed]. Basiliximab doses of between 0.001 and 10 µg/ml have been reported to cause marked CD25 blockade and thus reduction of lymphocyte proliferation when applied for up to 48 h to cell culture.^16,17^ Trials were performed using 0.01, 0.1, and 1.0 µg/ml basiliximab to sorted T_reg_ or T_eff_ in culture. Following the defined treatment period, cells were stimulated with 0–100 IU/ml IL-2 and the functional pSTAT5 assay was performed as described above. An untreated sample was used as a control. A 6-h application of 0.1 and 1.0 µg/ml basiliximab caused marked, but not complete, pSTAT5 suppression, and were taken forward into assays on patient samples.

### 2.7. **Assessment of T**_**reg**_**phenotype using CyTOF**

A Cytometry Time of Flight [CyTOF] mass cytometry technique was employed to allow in-depth multi-parameter assessment of the T_reg_ phenotype. Two panels [Supplementary Figure 3, available as Supplementary data at *ECCO-JCC* online] were designed to assess T_reg_ trafficking capacity [Panel 1] and T_reg_ suppressive ability [Panel 2]. The panels were extensively optimised and dose titration of each antibody was performed on PBMCs isolated from a blood bank cone, with all experiments performed on the same cone to minimise donor variability in marker expression. Antibodies were sourced from Fluidigm and have been validated for human use as per the manufacturer.

Cells for CyTOF staining were defrosted the day before the experiment and rested overnight in complete RPMI supplemented with 10% FCS at 37^°^C, 5% CO_2_. Cells were washed and dispersed into FACS staining tubes at 2 x 10^6^ cells per panel. A cisplatin viability stain was performed (cells re-suspended in 1 ml PBS, with 1 μl of cisplatin for 5 min, then reaction quenched with 3 ml MACS buffer, which was used as the cell staining medium [CSM] for all CyTOF experiments). Cells were then centrifuged at 400 x *g* for 5 min at room temperature, supernatant discarded, pellet dispersed, and 4 μl of FC block [MACS, Miltenyi Biotec] applied per sample. Conjugated antibodies were purchased from Fluidigm. Unconjugated antibodies were purchased from Biolegend and conjugated to the requisite metal isotopes using MaxPar labelling kits [Fluidigm]. A master mix of the extracellular CyTOF antibodies was made up for each panel and the samples were stained in a total volume of 100 μl for 30 min at room temperature. Following staining, cells were washed with CSM and re-suspended in fix/perm buffer [eBioscience FOXP3/Transcription factor staining buffer set] followed by a 1-h incubation at room temperature. Cells were washed with 1x permeabilisation buffer and stained with intracellular/transcription factor antibody master mix for 30 min at room temperature. Cells were then washed again with 1x permeabilisation buffer and re-suspended in 500 μl of 2% paraformaldehyde [PFA], left at room temperature for 1 h, and refrigerated overnight. On the day of acquisition, the cells were pelleted, washed, and resuspended in 1x Ir [Iridium] intercalator solution for 30 min; this acted as a DNA/final viability stain. After two further PBS washes, cells were re-suspended in water at a maximal concentration of 5 x 10^5^ with an internal bead standard [DVS EQ beads, Fluidigm].

### 2.8. Analysis of CyTOF data

Raw FCS files were normalised using Fluidigm CyTOF Software [https://www.fluidigm.com/software]. Data were uploaded to Cytobank [https://mrc.cytobank.org] and standard pre-processing performed to remove debris, doublets, beads, and dead cells. CD4^+^ T Cells were manually gated and used as a starting population for the automated unsupervised analysis.

Cytobank’s implementation of the t-Distributed Stochastic Neighbour Embedding [t-SNE] algorithm [known as viSNE]^18^ was used to transform the data to two dimensions, while still conserving the high dimensional structure of the data. The resulting t-SNE1 and t-SNE2 dimensions were then inputted into a clustering algorithm-spanning tree-progression analysis of density normalised events [SPADE; available on Cytobank],^19^ which extracts a cellular hierarchy from high-dimensional cytometry data and presents the populations in a branched tree diagram.

Cluster frequencies were quantified based on SPADE population clusters, and different cell populations were identified based on the median expression of known markers using our in-house developed pipeline [CytoClustR; https://github.com/kordastilab/cytoClustR]. For example, T_reg_ and T_eff_ nodes were identified in the SPADE tree based on the expression of CD127, CD25, and FOXP3. Further populations were identified within the T_reg_ and T_eff_ nodes, based on differential expression of one or more of the rest of the markers within those populations. Both marker expressions and cell abundances were analysed with one-way analysis of variance [ANOVA] for differences between the genotype groups for each identified population.

To quantify similarity/difference between the populations as well as expression of different markers, marker enrichment modelling [MEM] scores were calculated for each identified population and the output was visualised using heatmaps. The MEM algorithm objectively describes characteristics of cell populations using large numbers of markers, rather than a few most differentially expressed, and provides each marker with a score which denotes its expression level.^20^

### 2.9. TCR sequencing

#### 2.9.1. RNA extraction

1 x 10^5^ T_reg_ and T_eff_ were FACS-purified [BD Aria II] into TRIzol LS reagent [Invitrogen]. Total RNA was extracted as per the manufacturer’s instructions. To aid the precipitation of RNA, GlycoBlue™ Coprecipitant [Invitrogen] was used as per the manufacturer’s instructions. Processed samples were assessed for RNA integrity with an Agilent RNA 6000 Pico Kit in conjunction with an Agilent 2100 Bioanalyser. RNA concentration was quantified using a Qubit RNA HS Assay Kit [Invitrogen] and a Qubit analyser. Paired samples with RIN >9 were shipped to iRepetoire [iRepertoire Inc., Huntsville, AL] for TCR beta chain amplification and sequencing.

#### 2.9.2. Library construction procedure

For the construction of human TCR beta chain libraries, iRepertoire used amplicon rescued multiplex PCR [arm-PCR] to amplify each of the 18 submitted RNA samples. The cDNA synthesis protocol involved initial first-round reverse transcriptase [RT]-PCR using high concentrations of gene-specific primers [iRepertoire’s HTBI-M primers], followed by Beckman Coulter’s SPRIselect bead-cleanup procedure, then PCR2 using universal primers to amplify the cDNA exponentially.

### 2.10. Statistical analysis

Flow cytometric data were analysed with FlowJo 10.6.0 for MacOsX. Statistical analysis was performed with GraphPad Prism 8.4.1 for MacOsX. Normality was assessed using D’Agostino Pearson testing. Continuous data are presented as mean ± standard error of the mean for normally distributed variablesand as medians and interquartile ranges for skewed variables. Comparison of central tendency was performed using paired parametric and non-parametric tests as appropriate [t test or Wilcoxon signed rank test, respectively]. Similarly, multiple means [or ranks] were compared by one- or two-way ANOVA or Kruskal‐Wallis tests, as appropriate. For comparison of matched values, the Wilcoxon matched pairs signed rank test was used. The Mann‐Whitney test with a two-tailed *p*-value was used to determine significance level in all unmatched values. The TT, CT, and CC groups were matched by age and gender. For all significance testing, alpha was set to 0.05.

## 3. Results

### 3.1. rs61839660 modulates the expression of CD25 on CD4^+^ T cell subsets

We sought to investigate the effect of rs61839660 on CD25 cell surface expression, downstream signalling, and effector cell function. We obtained peripheral blood samples from genotyped CD patients and healthy controls, either homozygous minor allele [TT], homozygous major allele [CC], or heterozygous [CT], for rs61839660 from the National Institute of Health Research [NIHR] Inflammatory Bowel Disease BioResource,^21^ a UK-based biobank which holds demographic and genetic information pertaining to almost 50 000 IBD patients. Patient demographics are summarised in Table 1. There were no significant differences between genotype groups in terms of age, sex, or disease distribution. There was no difference in rates of medication use or need for surgery, suggesting that minor allele carriers do not have a more severe disease phenotype.

**Table 1.** NIHR IBD Bioresource Crohn’s disease patient demographics.

	TT [*n* = 14]	CT [*n* = 21]	CC [*n* = 22]	*p-*value
Age [mean]	50.2 ± 13.58	47.2 ± 13.40	52.1 ± 14.41	ns
Sex [% female]	57.1	57.1	36.4	ns
Disease distribution [% colonic disease]	71.4	33.3	36.8	ns
Disease distribution [% perianal disease]	35.7	19.0	18.2	ns
Biologic medication [% exposed]	7.7	30.8	15.4	ns
Surgery [% yes]	60.0	52.4	54.5	ns
Extraintestinal manifestations [% yes]	28.6	38.1	18.2	ns

Statistical analyses performed using one-way ANOVA for age and chi square analysis for categorical variables.

TT, homozygous minor allele; CT, heterozygous; CC, homozygous major allele, ns, not significant;ANOVA, analysis of variance.

Results considered significant if *p* </= 0.05.

**Figure 1.**
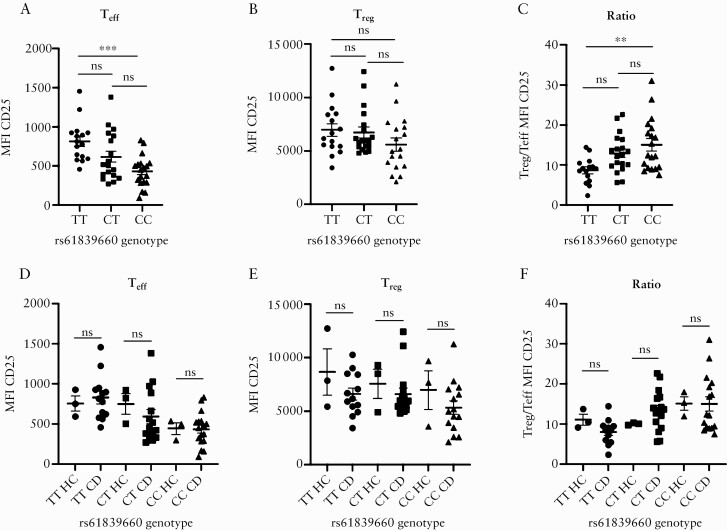
rs61839660 modulates CD25 expression on effector T cells. [a] Mean fluorescence intensity [MFI] of CD25 flow cytometry staining by genotype in CD4^+^ effector T cells [T_eff_]. T_eff_ from minor allele homozygotes [TT] demonstrated increased expression of CD25 compared with major allele homozygotes [CC] [*p* = 0.0002]. [b] MFI of CD25 staining by genotype in T_reg_. There was no difference in the MFI of CD25 by genotype in regulatory T cells [T_reg_]. [c] Ratio of CD25 expression in T_reg_/T_eff_. TT subjects demonstrated a reduced ratio of CD25 expression compared with CC subjects [*p* = 0.002]. [d] There was no difference in CD25 expression in T_eff_ or [e] T_reg_ or [f] in the MFI CD25 ratio T_reg_/T_eff_ between CD patients and healthy controls within each genotype group. Mean ± SEM plotted. Statistical analyses performed using one-way ANOVA, Tukey post hoc test, for comparisons involving more than two groups, and unpaired t- ests for comparisons involving two groups. TT: *n* = 16, CT: *n* = 19, CC: *n* = 19. **p <0.01, ***p <0.001. HC, healthy control; CD, Crohn’s disease; ns, not significant; SEM, standard error of the mean; ANOVA, analysis of variance.

CD4^+^ T_reg_ and T_eff_ were sorted by flow cytometry [Supplementary Figure 1]. We demonstrated increased CD25 cell surface expression in CD patients and healthy controls, specifically on T_eff_ of TT patients as measured by mean fluorescence intensity [MFI] of flow cytometry staining [Figure 1a], compared with CC subjects. There was a non-significant trend for increased CD25 expression on T_reg_ in TT patients [Figure 1b] as well as a non-significant trend towards enhanced CD25 expression on T_eff_ and T_reg_ from CT heterozygote carriers of the minor allele at rs61839660 [Figure 1a, b, and d]. The ratio of T_reg_:T_eff_ CD25 expression was significantly reduced in TT patients [Figure 1c] compared with CC patients. No difference was seen in T_reg_ or T_eff_ CD25 expression between CD patients and healthy controls with the same genotype [Figure 1d–f], suggesting that differential CD25 expression between genotypes is conferred by the risk allele, rather than by the presence of inflammatory disease.

### 3.2. rs61839660 regulates responsiveness of CD4^+^ T cells to IL-2 signalling through altered CD25 expression

**Figure 2.**
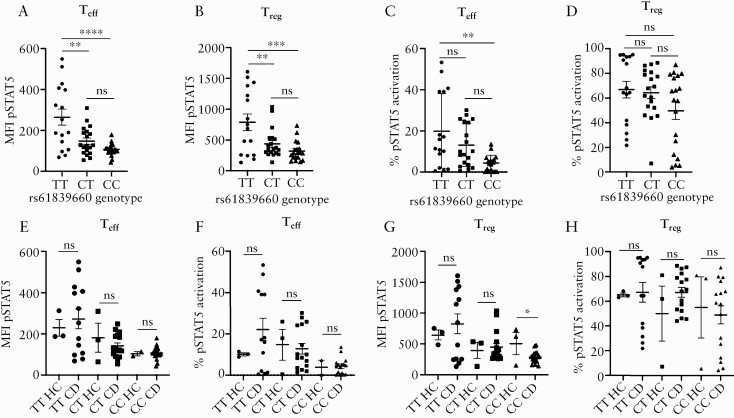
STAT5 phosphorylation in response to IL-2 is enhanced in carriers of the rs61839660 minor allele. Signal transducer and activator of transcription 5 [STAT5] phosphorylation [pSTAT5] was assessed after 15 min of incubation with 10 IU/ml of recombinant human IL-2 in CD4^+^ effector T cells [T_eff_] and regulatory T cells [T_reg_]. [a] Minor allele homozygotes [TT] demonstrated increased mean fluorescence intensity [MFI] for pSTAT5 flow cytometry staining compared with heterozygous [CT] and major allele homozygous [CC] subjects in T_eff_ [TT vs CC *p* <0.0001; TT vs CT *p* = 0.0026] and [b] T_reg_ [TT vs CC *p* = 0.0004; TT vs CT [*p* = 0.0085]. [c] The proportion of cells with pSTAT5 staining was also increased in T_eff_ from TT subjects compared with CC subjects [*p* = 0.0012]. [d] No differences were detected in T_reg_ when comparing the percentage of pSTAT5 stained cells between the genotypes. [e] There was no difference in pSTAT5 staining MFI or [f] proportion of pSTAT5-positive cells in T_eff_ or [g‐h] T_reg_ between healthy controls [HC] and Crohn’s disease [CD] patients in carriers of the minor allele. Mean ± SEM plotted. Statistical analyses performed using one-way ANOVA, Tukey post hoc test, for comparisons involving more than two groups and unpaired t tests for comparisons involving two groups. TT: *n* = 16, CT: *n* = 19, CC: *n* = 19. *p <0.05, ** *p* <0.01, ***p <0.001, ****p <0.0001. SEM, standard error of the mean; ANOVA, analysis of variance; ns = not significant.

To determine whether rs61839660 affected the CD4^+^ T cell response to IL-2 signalling, the extent of STAT5 phosphorylation [pSTAT5] was measured in T_eff_ and T_reg_ following short-term exposure to 10 IU IL-2 [Figure 2]. In optimisation work, this concentration of IL-2 was found to be sufficient to induce pSTAT5 expression in approximately 50% of T_reg,_ with no pSTAT5 expression in T_eff_ in CC patients. T_eff_ and T_reg_ from TT patients had an enhanced pSTAT5 response as measured by mean fluorescence intensity [MFI] of pSTAT5, compared with CT and CC patients [Figure 2a and b]. T_eff_ from TT subjects also exhibited a higher proportion of pSTAT5^+^ cells compared with T_eff_ from CC patients [Figure 2c]. There was a non-significant increase in the proportion of pSTAT5^+^ T_reg_ in TT patients [Figure 2d]. There was no difference in T_eff_ or T_reg_ pSTAT5 response to IL-2 signalling between healthy controls and CD patients of the same genotype [Figure 2e, f, g, and h]. As noted for CD25 expression, a non-significant trend for enhanced pSTAT5 staining was observed in CT heterozygote carriers of the minor allele, and this was more apparent in T_eff_ than T_reg_ [Figure 2a–h].

It was noted that among TT subjects, there was a subgroup that exhibited a more pronounced pSTAT5 response to IL-2 in both T_eff_ and T_regs_ [Figure 2a–d]. We performed a subanalysis on this ‘hyper-responder’ cohort, defining subjects with a T_eff_ MFI pSTAT5 greater than 395 and a T_reg_ MFI pSTAT5 greater than 1100 in response to 10 IU/ml IL-2, as ‘hyper-responders’ [Table 2]. The same five subjects with hyper-responsive T_eff_ also exhibited an exaggerated T_reg_ pSTAT5 response. There was no significant difference in age, sex, disease distribution, disease severity, or proportion of patients exposed to biologic medication between the TT ‘hyper-responders’ and the remaining TT subjects. There was no significant difference in T_eff_ or T_reg_ CD25 expression between the TT ‘hyper-responder’ subgroup and the remaining TT subjects.

**Table 2.** Characteristics of the TT ‘hyper-responder’ cohort.

	TT Hyper-responder [n = 5]	TT Other [n = 11]	*p* value
Mean T_eff_ CD25 MFI	795.4	825.2	ns
Mean T_reg_ CD25 MFI	5924	7631	ns
Mean T_reg_/T_eff_ CD25 MFI	7.46	10.94	ns [0.076]
Age [mean]	33.6	39.9	ns
Sex [% female]	100.0	72.7	ns
Disease distribution [% colonic disease]	20.0	50.0	ns
Disease distribution [% perianal disease]	66.6	60.0	ns
Biologic medication [% exposed]	40.0	12.5	ns
Surgery [% yes]	100.0	60.0	ns

Statistical analyses performed using unpaired t test to compare means and chi square analysis for categorical variables.

TT, homozygous minor allele; ns, not significant.

Results considered significant if *p* </= 0.05.

As a control, the pSTAT5 response to IL-7 was examined across a range of doses [0‐1000 IU]. rs61839660 did not affect CD127 surface expression across the genotypes in T_eff_ or T_reg_ [Supplementary Figure 2a and b, available as Supplementary data at *ECCO-JCC* online]. Importantly, STAT5 phosphorylation downstream of IL-7 signalling was independent of genotype in T_eff_ or T_reg_ [Supplementary Figure 2c–h], indicating that the differences in pSTAT5 response to IL-2 stimulation are likely caused by enhanced CD25 expression in the presence of the minor allele.

### 3.3. **The rs61839660 minor allele enhances transcription of pro-inflammatory cytokines following exogenous IL-2 and acute TCR stimulation of T**_**effs**_

Together, our findings suggested that the rs61839660 minor allele may confer a functional ‘hyper-responsive’ state to IL-2 signalling in T_eff_. To further investigate this proposal, we sought to identify whether presence of the minor allele influenced the functional response to IL-2 by T_eff_, by measuring cytokine expression [*IFNG* and *TNF*] responses by the cells after TCR stimulation.

T_eff_ from cryopreserved PBMCs of CD patients, genotyped as CC and CT at rs61839660, were isolated by FACS and used to conduct the assays. T_eff_ were stimulated for 24 h with a range of IL-2 doses only, or with IL-2 followed by a brief stimulation [6 h] with different concentrations of plate-bound αCD3/αCD28 [Figure 3].

We observed that for both expression of *IFNG* [Figure 3a and b] and *TNF* [Figure 3c and d], compared with CC samples, the presence of the minor T allele at rs61839660 enhanced the relative transcript abundance in response to IL-2, together with TCR stimulation. This was most clearly observed at the highest concentration of IL-2 [100 IU/ml] for each of the TCR cross-linking conditions [Figure 3a and c]. Importantly, greater pro-inflammatory cytokine relative transcript abundance was also observed in CT samples treated with 100 IU/ml of rhIL-2 in the absence of TCR crosslinking, despite similar baseline [0 IU IL-2, no TCR cross-linking] transcript abundance levels between the genotypes [Figure 3b and d]. These data support the notion that within the context of CD, presence of the rs61839660 minor allele confers a functional capacity for hyper-responsiveness of CD4^+^ T_eff_ in response to IL-2 signalling.

### 3.4. **CD25 blockade is effective in suppressing the enhanced pSTAT5 response conferred by the rs61839660 minor allele**

Basiliximab, a mouse-human chimeric monoclonal antibody which antagonises CD25, is currently licensed for the prevention of renal allograft rejection. We examined whether basiliximab added to *in vitro* culture of sorted T_reg_ and T_eff_ could block pSTAT5 activation in a cohort of CD patients with CC and CT rs61839660 genotypes, recruited from our local patient population. Basiliximab was effective in blocking T_eff_ and T_reg_ pSTAT5 activation in both genotypes [Figure 4a–f], confirming that the enhanced pSTAT5 response is dependent on signalling through CD25. Of note, CT T_eff_ also demonstrated elevated baseline pSTAT5 activation in the absence of basiliximab, when compared with CC T_eff_ [Figure 4d], as previously seen in the other sample cohorts used in our study. There was no significant difference in the response to basiliximab between the genotype groups.

### 3.5. **T**_**eff**_**in subjects homozygous for rs61839660 demonstrate an activated, gut-homing phenotype**

**Figure 5.**
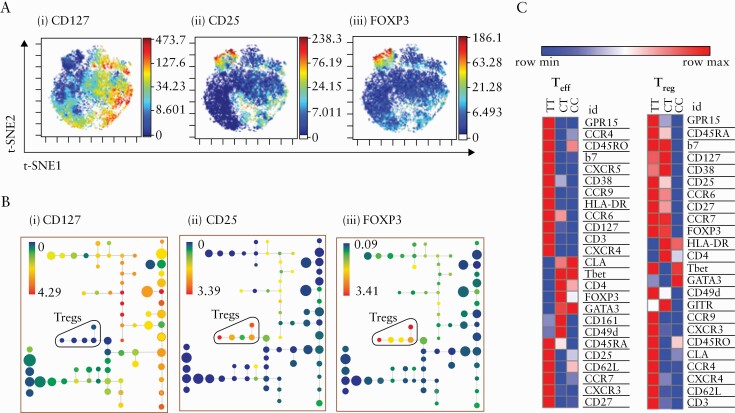
CD4^+^ T cell effectors of minor allele homozygotes at rs61839660 demonstrate a more activated gut-homing phenotype. [a] Identifying regulatory T cells [T_reg_] and non-Tregs [CD4^+^ T effectors, T_eff_] using viSNE map. Cell population islands coloured by expression of [i] CD127, [ii] CD25, [iii] FOXP3. Red-blue colouring denotes highest to lowest level of expression. [b] Identifying T_reg_ and T_eff_ using SPADE [Spanning-tree Progression Analysis of Density-normalised Events] trees. Each node denotes a cluster. Proximity to other nodes denotes expression of similar markers. T_reg_ populations are coloured by the expression of [i] CD127, [ii] CD25, [iii] FOXP3. Red-blue colouring denotes highest to lowest level of expression. [c] Marker Enrichment Modelling [MEM] analyses of T_eff_ for rs61839660 minor allele homozygote [TT], heterozygote [CT], and major allele homozygote [CC] Crohn’s disease patient groups. Scaling is done per row [red = row maximum, blue = row minimum]. Higher MEM score denotes higher expression of a marker in a group, compared with another.

To further elucidate the phenotype of T_reg_ and T_eff_ from patients harbouring the rs61839660 minor variant, we performed an in-depth phenotypic analysis using Cytometry Time of Flight [CyToF] [Panels 1 and 2 and antibody details in Supplementary Figure 3], with unsupervised clustering analysis and Marker Enrichment Modeling [MEM].^20^ T_reg_ and T_eff_ subsets were identified by CD25, CD127, and FOXP3 viSNE plots [Figure 5a] and SPADE clustering analysis [Figure 5b] and analysed with specific focus on the expression of trafficking and cell activation markers. The number of cells per each SPADE cluster and the T_eff_ and T_reg_ groups per subject were recorded, and tables for the T_eff_ and T_reg_ groups for Panel 1 and Panel 2 may be viewed in Supplementary Data Files 1 and 2, respectively, available as Supplementary data at *ECCO-JCC* online. The dimensionality reduction was performed in Cytobank with the following setup parameters: equal sampling of 22 500 [Panel 1] and 29 000 [Panel 2] CD4^+^ cells per subject, perplexity = 30, theta = 0.5, and iterations = 5000. Each group was composed of 10 subjects. The resulting t-SNE1 and t-SNE2 dimensions of the viSNE transformation were used as input for the SPADE clustering. The following markers were used for the viSNE analysis for Panel 1: CCR6, CLA, CD31, CD49d, CD62L, CD161, GITR, FOXP3, b7, CCR7, CD127, T-bet, GATA3, CCR9, CD25, CXCR5, CD38, CXCR4, HLA-DR, and for Panel 2: OX40, PD1, LAG3, ICOS, CD62L, CD137, CD161, CD39, Ki67, FOXP3, CD30, PDL1, CD127, T-bet, GATA3, CD37, CD25, CTLA-4, Granzyme-B, CD120b, HLA-DR, and Perforin.

Confirming observations made by flow cytometry, TT T_eff_ demonstrated increased CD25 expression [Figure 5a]. However, this analysis also revealed a comparable increase in CD25 expression on TT T_reg_, providing an explanation as to why homozygous minor allele T_regs_ also exhibited hyper-responsiveness during IL-2/STAT5 phosphorylation assays [Figure 2b]. Furthermore, this analysis demonstrated that both T_reg_ and T_eff_ from minor allele homozygotes have a more activated, gut-homing phenotype compared with cells from CT and CC subjects [Figure 5c]. Key markers that were differentially upregulated in TT patients included the chemokine receptor CXCR3, which is typically associated with inflammatory Th1 cell infiltration, and CXCR4, associated with IL-2-mediated immune activation. GPR15, which specifically traffics cells to the colon, was also preferentially expressed on both T_reg_ and T_eff_ of TT patients, as were other markers associated with T_reg_ tissue trafficking such as CCR6 and integrin alpha 7 [Supplementary Figures 4a–c and 5a–d, available as Supplementary data at *ECCO-JCC* online]. In addition, T_eff_ from the TT group were the only cells to express Ki67 [Supplementary Figure 5c], indicating an enhanced proliferative effector phenotype and cellular activation.

### 3.6. TT patients may have a reduced capacity to mount a regulatory T cell response

**Figure 6.**
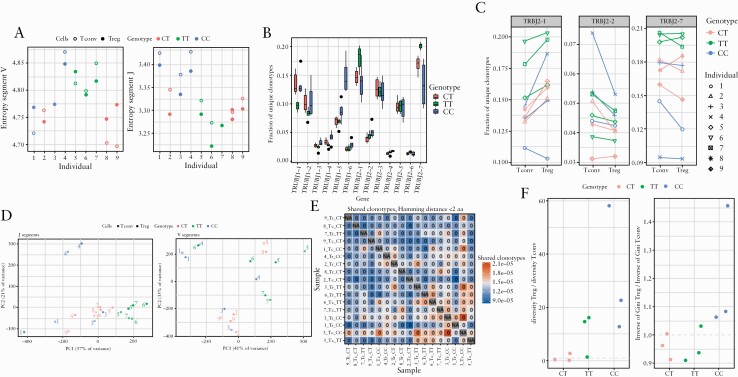
T cell receptor profiling reveals that rs61839660 minor allele homozygote Crohn’s disease patients may have a reduced capacity to mount a regulatory T cell response. [a] CDR3 region J and V segment entropy for CD4^+^ effector T cells [T_eff,_ empty circles] and regulatory T cells [T_reg,_ filled circles] among the genotypes. [b] Fractions of unique J segment clonotypes used by each genotype. [c] TT patient CD4^+^ T cells (T_reg_ and T_eff_ /conventional [T_conv_] cells) preferentially use two TRBJ segments [TRBJ2-1 and TRBJ2-7]. T_reg_ and T_eff_/T_conv_ from the same patient are denoted by conjoined lines and matching individual symbols. [d] Principal component analysis of J segment usage revealed distinct clustering by genotype.[ e] Heatmap for clustering of shared clonotypes matched by Hamming distance. [f]: TCR diversity ratio for T_reg_/T_eff_, measured by Gini index. TCR, T cell receptor.

To assess whether the difference in phenotypes observed between the three patient groups was driven by their ability to respond to antigens, we performed T cell receptor [TCR] sequencing of T_reg_ and T_eff_ from three CD patients from each SNP genotype. We assessed the variability displayed in both the V and J segment of the CDR3 region. We found that the J segments in TT patient cells consistently displayed the least variability, those from CC patient cells displayed the greatest variability, and the CT patients had an intermediate phenotype [Figure 6a]. J segments in T_eff_ showed higher variability than T_reg_ across the three genotypically defined groups. Further analysis of individual J segments revealed that TT cells preferentially use two TRBJ segments, segments TRBJ2-1 and TRBJ2-7 [Figure 6b and c]. Principal component analysis of J segment usage revealed distinct clustering by genotype [Figure 6d]. Such differential clustering was not observed on analysis of the V segment usage. We then examined whether any of the individuals cluster together based on shared clonotypes on either an amino acid or a nucleotide level, and did not observe clustering based on genotypes [Figure 6e]. Finally, to assess whether TCR diversity was different between T_reg_ and T_eff_ subsets, we examined their diversity ratio across the three genotypes. We found that this ratio was lower in TT and CT patients compared with CC patients. Whereas T_regs_ were more diverse than T_eff_ [measured by Gini index] in CC patients, in TT and CT patients, T_reg_ diversity was comparable to or lower than T_eff_ [Figure 6f]. The reduced T_reg_ diversity when compared with T_eff_ in TT patients, coupled with T_eff_ hyper-responsiveness to IL-2, may suggest that TT patients are less efficient at mounting a regulatory response to counter T_eff_-driven inflammation.

In summary, we show that the rs61839660 minor allele enhances sensitivity to IL-2 signalling in CD4^+^ T cells by increasing CD25 expression. We propose that the reduced ratio of T_reg_:T_eff_ CD25 expression in individuals homozygous [TT] for this SNP may increase the ability of T_eff_ to respond to low environmental IL-2, permitting activation of T_eff_ at levels of IL-2 which would usually only activate T_reg_. This T_eff_ hyper-responsiveness to IL-2 could therefore be a major driver of inflammation in these subjects. Mass cytometric analysis of immune cells from these patients confirmed that CD25 is elevated in patients homozygous for the risk allele at rs61839660, and that effector T cells from these patients are primed to home to the intestine by virtue of upregulation of gut trafficking molecules such as alpha 4 beta 7 integrin and GPR15.

## 4. Discussion

Here we show that rs61839660 enhances IL-2 signalling in CD4^+^ T cells from CD patients by regulating the expression of CD25. Effector T cells from TT patients display the ability to respond to doses of IL-2 that normally only activate regulatory T cells. Additionally, T_eff_ from carriers of the minor allele express elevated transcript levels of pro-inflammatory cytokines in response to IL-2 and/or TCR signalling—supportive of our conclusion that rs61839660 regulates the functional responsiveness of T cells in CD patients via the regulation of sensitivity to IL-2 signalling. Our findings are in contrast to previous work involving CRISPR inactivation of immune enhancers in mice, which proposed that rs61839660 impaired induction of CD25 and therefore disrupted IL-2 signalling in T cells, and found that carriage of the risk allele was associated with reduced CD25 transcript levels in stimulated undifferentiated CD4^+^ T cells from healthy human subjects.^15^ However, our findings are supported by a previous study of 323 healthy human subjects, which found that the presence of the rs61839660 minor allele was associated with increased *IL2RA* transcript levels and surface expression of CD25 on CD4^+^ memory T cells.^14^

Basiliximab was found to effectively block T_eff_ hyper-responsiveness in minor allele heterozygote [CT] patients. Previous clinical trials of basiliximab in small cohorts of unselected patients with ulcerative colitis, in whom rs61839660 had not been identified as a risk locus, showed safety but did not demonstrate efficacy compared with conventional treatment.^22,23^ However, these data suggest that basiliximab could have a role in blocking the hyper-responsive T_eff_ population seen in rs61839660 carriers. Further *in vitro* experimental work is required to assess the potential therapeutic value of CD25 blockade in this cohort.

Mass cytometric analysis of immune cells from these patients was also performed, which confirmed that CD25 expression is elevated in patients homozygous for the TT allele at rs61839660 and that effector T cells from these patients are primed to home to the intestine by virtue of upregulation of gut-trafficking molecules such as alpha 4 beta 7 integrin and GPR15. Analysis of the TCR repertoire further reveals that diversity in the T_reg_ subset does not mirror diversity in the T_eff_ subset in TT patients. Lack of ability in the T_reg_ compartment to respond to aberrant T_eff_ responses in TT patients, may be another underlying pathogenic mechanism. Unlike in type 1 diabetes mellitus, where rs61839660 is protective,^12,14^ in CD rs61839660 is linked to pathology. Based on our observations, the mechanism of this likely involves the capacity of rs61839660 to render T_eff_ hyper-responsive to doses of IL-2 which typically activate only T_regs_, putatively resulting in an enhanced activation profile of the cells. This, alongside an activated gut-homing profile of TT T_eff_, potentially explains the association between rs61839660 and CD. However, it will be necessary to follow up our observational analyses with further mechanistic studies to fully confirm or refute these hypotheses.

This study provides a unique perspective on the link between genetic risk and the development of disease, by studying genotyped CD and HC patients recruited across the UK. Recruiting genotyped human subjects across the country presents a logistically complex process; thus a limitation of our study is that insufficient patient numbers could be recruited to adequately study the link between disease severity and the risks allele. Further studies are also needed to define the interplay between the rs61839660 risk allele and other genotypic and environmental factors in patients with CD, to allow for a precise definition of the mechanism of pathogenicity of the rs61839660 risk allele.

This is one of the first works in IBD to link a pathogenic mechanism to a CD-associated genetic variant, advancing the goal of a personalised therapeutic approach. Accurate phenotypic and genetic profiling of CD patients would allow targeted therapeutic intervention, permitting selection of a treatment targeted to correct the aberrant pathway in a given patient. We highlight the vital role of open-access resources, such as the NIHR IBD BioResource, for sourcing genetically selected material for functional analysis of rare variants. We provide a novel mechanistic link between genotype and function relevant to multiple human autoimmune diseases. Over 40 phase 2/3 trials of low-dose IL-2 in the treatment of autoimmune conditions are currently registered, including in CD [ClinicalTrials.gov NCT01988506]. These trials typically use doses of 0.5‐3 x 10^6^ IU/m^2^ body area, which are delivered subcutaneously under trial-specific regimens. The premise of this treatment is that low doses of IL-2 will preferentially expand the tolerising T_reg_ population, correcting the dysregulated autoimmune response. However, given the exaggerated T_eff_ response to low doses of IL-2 in carriers of rs61839660, these subjects could be at increased risk of deleterious clinical outcomes due to theoretical T_eff_ hyperactivation. With 9.4% CD patients carrying the risk allele, this represents a significant subset of potential trial participants. Furthermore, our findings suggest there is merit in further assessing the role of basiliximab in genetically stratified patients, as an adjunct to existing treatments.

## Supplementary Material

jjab103_suppl_Supplementary_Data_S1

jjab103_suppl_Supplementary_Data_S2

jjab103_suppl_Supplementary_Figure_S1-S5

## References

[1] Jostins L , Ripke S, Weersma RK, et al; International IBD Genetics Consortium [IIBDGC]. Host-microbe interactions have shaped the genetic architecture of inflammatory bowel disease. Nature 2012;491:119–24. 23128233 10.1038/nature11582 PMC3491803

[2] Murthy SK , Begum J, Benchimol EI, et al Introduction of anti-TNF therapy has not yielded expected declines in hospitalisation and intestinal resection rates in inflammatory bowel diseases: a population-based interrupted time series study. Gut 2020;69:274–82. 31196874 10.1136/gutjnl-2019-318440 PMC6984056

[3] Noor NM , Verstockt B, Parkes M, Lee JC. Personalised medicine in Crohn’s disease. Lancet Gastroenterol Hepatol 2020;5:80–92. 31818474 10.1016/S2468-1253(19)30340-1

[4] Maul J , Loddenkemper C, Mundt P, et al Peripheral and intestinal regulatory CD4+ CD25[high] T cells in inflammatory bowel disease. Gastroenterology 2005;128:1868–78. 15940622 10.1053/j.gastro.2005.03.043

[5] Fantini MC , Pallone F, Monteleone G. Common immunologic mechanisms in inflammatory bowel disease and spondylarthropathies. World J Gastroenterol 2009;15:2472–8. 19468997 10.3748/wjg.15.2472 PMC2686905

[6] Farh KKH , Marson A, Zhu J, et al Genetic and epigenetic fine mapping of causal autoimmune disease variants. Nature 2015;518:337–43. 25363779 10.1038/nature13835 PMC4336207

[7] Fontenot JD , Gavin MA, Rudensky AY. Pillars article: Foxp3 programs the development and function of CD4+CD25+ regulatory T cells. J Immunol 2017;198:986–92. 28115587

[8] Hori S , Nomura T, Sakaguchi S. Pillars article: control of regulatory T cell development by the transcription factor Foxp3. J Immunol 2017;198:981–5. 28115586

[9] Liu W , Putnam AL, Xu-Yu Z, et al CD127 expression inversely correlates with FoxP3 and suppressive function of human CD4+ T reg cells. J Exp Med 2006;203:1701–11. 16818678 10.1084/jem.20060772 PMC2118339

[10] Boyman O , Sprent J. The role of interleukin-2 during homeostasis and activation of the immune system. Nat Rev Immunol 2012;12:180–90. 22343569 10.1038/nri3156

[11] Liu JZ , van Sommeren S, Huang H, et al; International Multiple Sclerosis Genetics Consortium; International IBD Genetics Consortium. Association analyses identify 38 susceptibility loci for inflammatory bowel disease and highlight shared genetic risk across populations. Nat Genet 2015;47:979–86. 26192919 10.1038/ng.3359 PMC4881818

[12] Huang H , Fang M, Jostins L, et al Fine-mapping inflammatory bowel disease loci to single-variant resolution. Nature 2017;547:173–8. 28658209 10.1038/nature22969 PMC5511510

[13] Jones GR , Lyons M, Plevris N, et al IBD prevalence in Lothian, Scotland, derived by capture-recapture methodology. Gut 2019;68:1953–60. 31300515 10.1136/gutjnl-2019-318936 PMC6839733

[14] Rainbow DB , Pekalski M, Cutler AJ, et al A rare IL2RA haplotype identifies SNP rs61839660 as causal for autoimmunity. bioRxiv February 2017:108126. doi:10.1101/108126.

[15] Simeonov DR , Gowen BG, Boontanrart M, et al Discovery of stimulation-responsive immune enhancers with CRISPR activation. Nature 2017;549:111–5. 28854172 10.1038/nature23875 PMC5675716

[16] Kircher B , Lätzer K, Gastl G, Nachbaur D. Comparative in vitro study of the immunomodulatory activity of humanized and chimeric anti-CD25 monoclonal antibodies. Clin Exp Immunol 2003;134:426–30. 14632747 10.1111/j.1365-2249.2003.02324.x PMC1808889

[17] Wang Z , Shi BY, Qian YY, Cai M, Wang Q. Short-term anti-CD25 monoclonal antibody administration down-regulated CD25 expression without eliminating the neogenetic functional regulatory T cells in kidney transplantation. Clin Exp Immunol 2009;155:496–503. 19141125 10.1111/j.1365-2249.2008.03847.x PMC2669526

[18] Amir el-AD , Davis KL, Tadmor MD, et al viSNE enables visualization of high dimensional single-cell data and reveals phenotypic heterogeneity of leukemia. Nat Biotechnol 2013;31:545–52. 23685480 10.1038/nbt.2594 PMC4076922

[19] Qiu P , Simonds EF, Bendall SC, et al Extracting a cellular hierarchy from high-dimensional cytometry data with SPADE. Nat Biotechnol 2011;29:886–91. 21964415 10.1038/nbt.1991 PMC3196363

[20] Diggins KE , Greenplate AR, Leelatian N, Wogsland CE, Irish JM. Characterizing cell subsets using marker enrichment modeling. Nat Methods 2017;14:275–8. 28135256 10.1038/nmeth.4149 PMC5330853

[21] Parkes M ; IBD BioResource Investigators. IBD BioResource: an open-access platform of 25 000 patients to accelerate research in Crohn’s and Colitis. Gut 2019;68:1537–40. 31270165 10.1136/gutjnl-2019-318835 PMC6709775

[22] Creed TJ , Probert CS, Norman MN, et al; BASBUC Investigators. Basiliximab for the treatment of steroid-resistant ulcerative colitis: further experience in moderate and severe disease. Aliment Pharmacol Ther 2006;23:1435–42. 16669958 10.1111/j.1365-2036.2006.02904.x

[23] Sands BE , Sandborn WJ, Creed TJ, et al Basiliximab does not increase efficacy of corticosteroids in patients with steroid-refractory ulcerative colitis. Gastroenterology 2012;143:356–64.e1. 22549092 10.1053/j.gastro.2012.04.043

