# Supplementary material for: A Crohn’s Disease-associated IL2RA Enhancer Variant Determines the Balance of T Cell Immunity by Regulating Responsiveness to IL-2 Signalling
Source: J Crohns Colitis. 2021 Jun 12;15(12):2054–65. doi: 10.1093/ecco-jcc/jjab103 (PMC8684452; doi:10.1093/ecco-jcc/jjab103)
Supplement: jjab103_suppl_Supplementary_Figure_S1-S5 [file jjab103_suppl_supplementary_figure_s1-s5.docx]

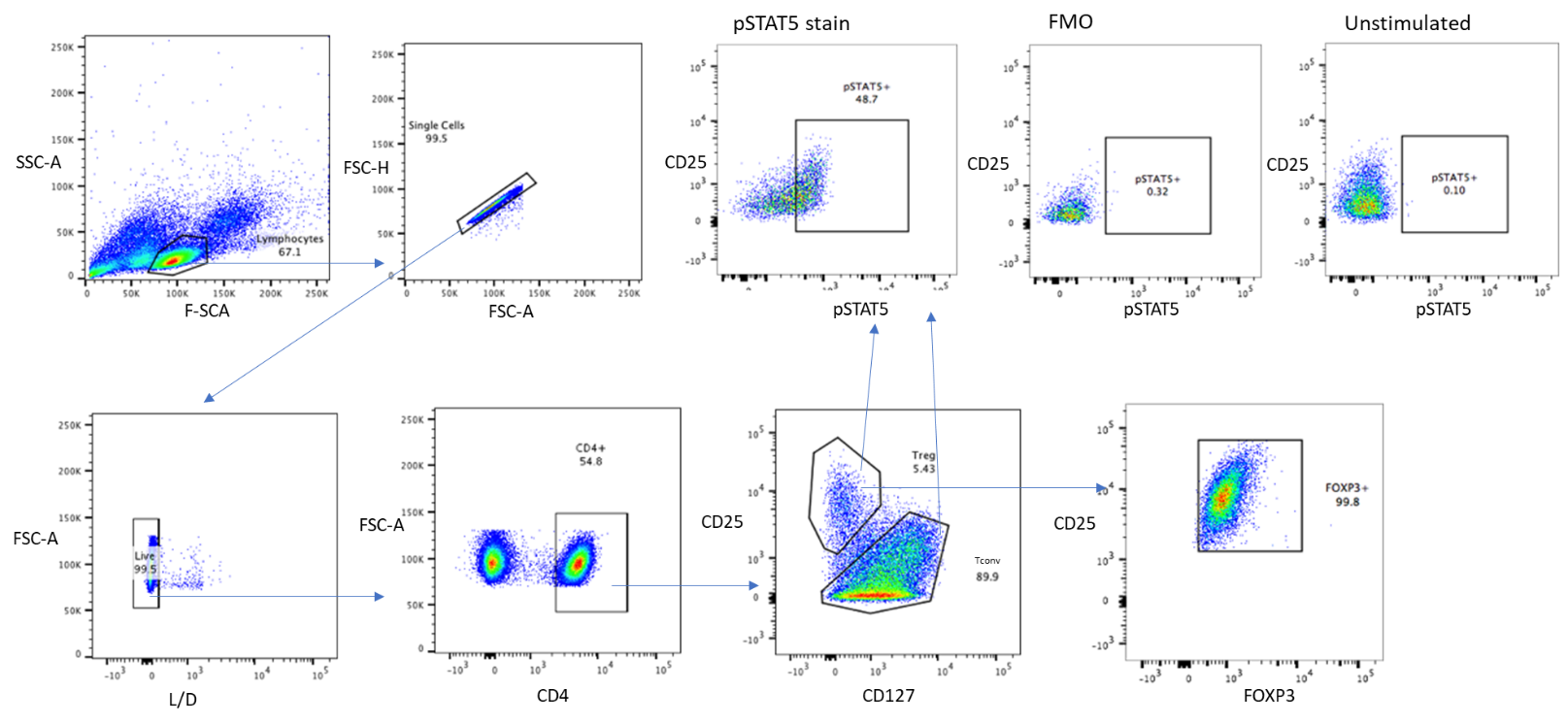


**Supplemental Figure 1: Gating strategy to define regulatory and CD4+ effector T cells and pSTAT5^+^ populations.** Fluorescence activated cell sorting (FACS) was performed on peripheral blood mononuclear cell collected from genotyped Crohn’s Disease patients identified through the National Institute of Health Research Inflammatory Bowel Disease Bioresource. Regulatory T cells (T_reg_) were sorted as CD4^+^CD25^high^CD127^low^ cells. CD4+ effector T cells were sorted as the CD4^+^CD25^int/low^CD127^+^ population. The population sorted as T_reg_ were highly positive for FOXP3, confirming their identity as T_reg_. Purified T_reg_ and T_eff_ were stimulated with 10 IU/ml recombinant human IL2 for 15 minutes. Following this, staining for phosphorylated signal transducer and activator of transcription 5 (pSTAT5) was performed. Flow cytometry gates for pSTAT5^+^ cells were set using fluorescence minus one (FMO) controls for pSTAT5 and unstimulated (no IL-2 treatment) cell samples.


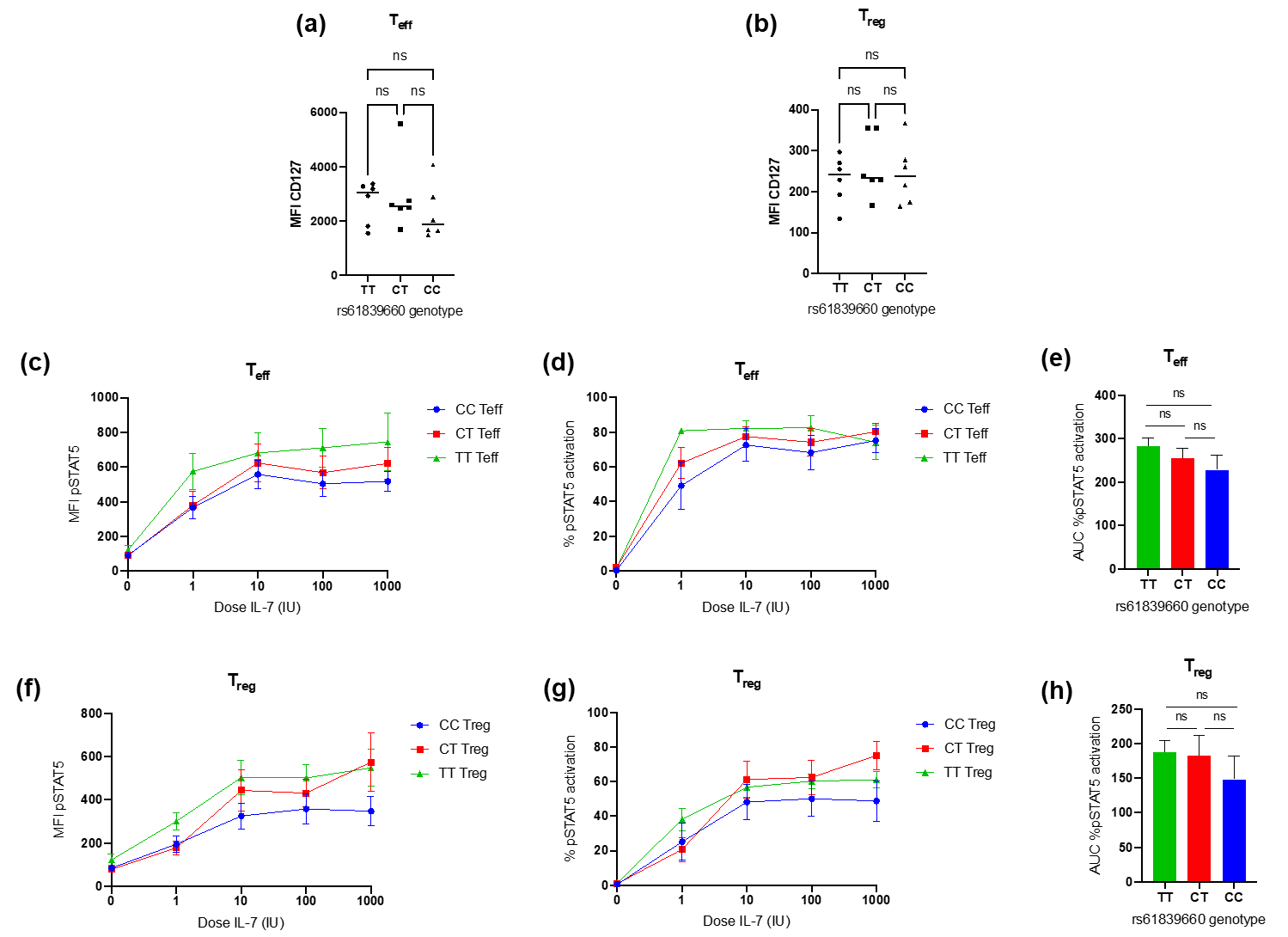


**Supplemental Figure 2: CD127 expression and STAT5 phosphorylation in response to IL-7 are not affected by rs61839660.** CD127 expression and signal transducer and activator of transcription 5 (pSTAT5) phosphorylation was assessed in CD4^+^ effector T cells (T_eff_) and regulatory T cells (T_reg_) from heterozygous (CT), major allele homozygous (CC) and minor allele homozygous (TT) subjects after 15 minutes incubation with different concentrations of recombinant human IL-7. **a:** There was no difference in the mean fluorescence intensity (MFI) for CD127 flow cytometry staining in T_eff_ and (**b**) T_reg_ between the three genotypes. **c:** pSTAT5 staining MFI and (**d**) proportion (%) of pSTAT5^+^ cells after stimulation with IL-7. **e**: No differences were observed in the area under curve (AUC) analysis in T_eff_ between the genotypes in response to IL-7. **f**: T_regs_ from CT, CC and TT subjects demonstrated a similar pSTAT5 response to IL-7 as shown by staining MFI and (**g**) % of pSTAT5^+^ cells, with no statistical differences between the groups. **h**: AUC analysis revealed no differences between the three genotypes after IL-7 stimulation. Mean ± SEM plotted. Statistical analyses performed using two-way ANOVA, Tukey’s multiple comparisons test for comparisons involving more than two groups and unpaired t-tests for comparisons involving two groups. AUC = area under curve, ns = not significant. TT (n=6), CT (n=6), CC (n=6).


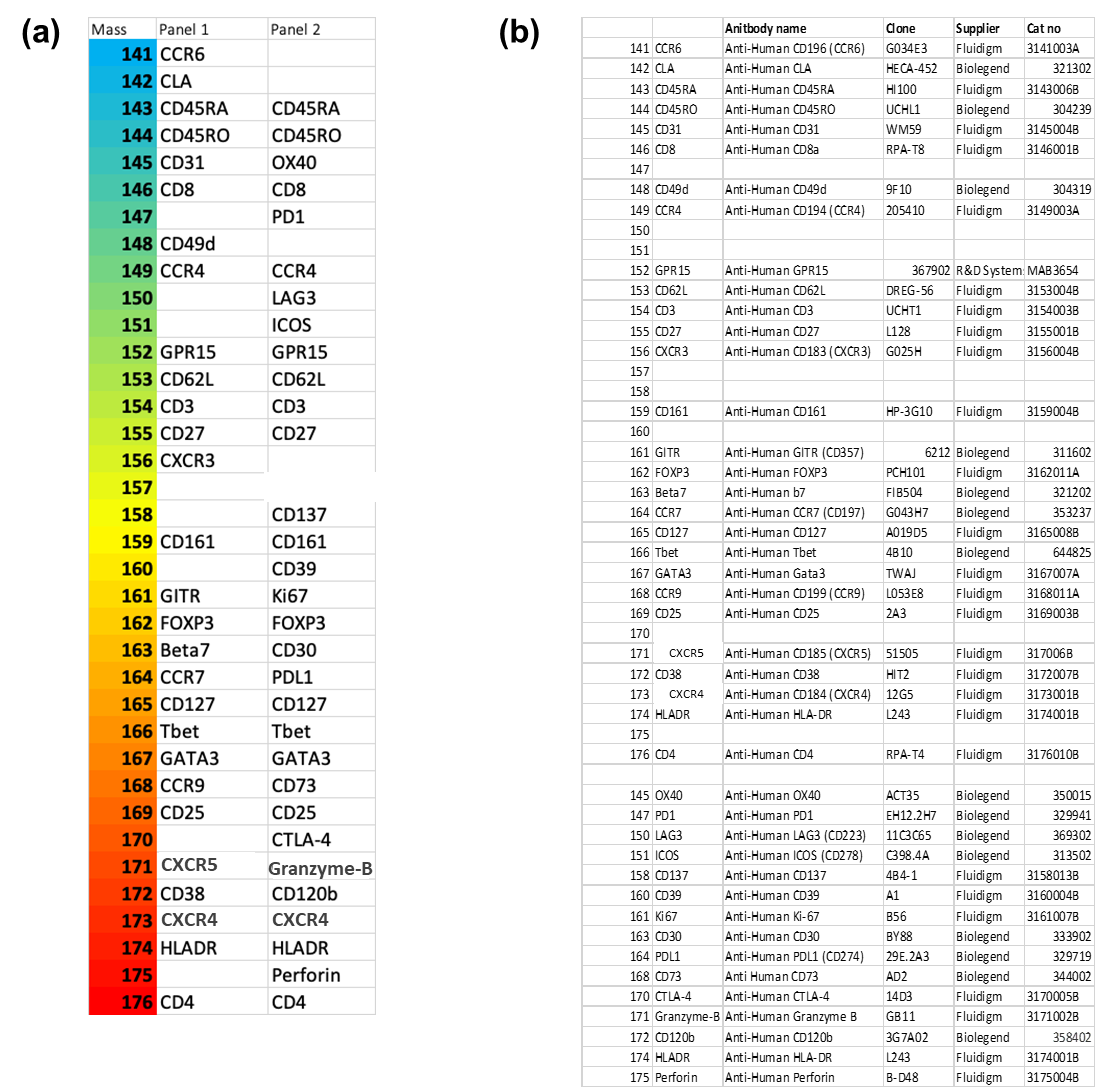


**Supplemental Figure 3: (a) Panels and (b) antibodies used for Cytometry Time of Flight (CyTOF) analysis**

**
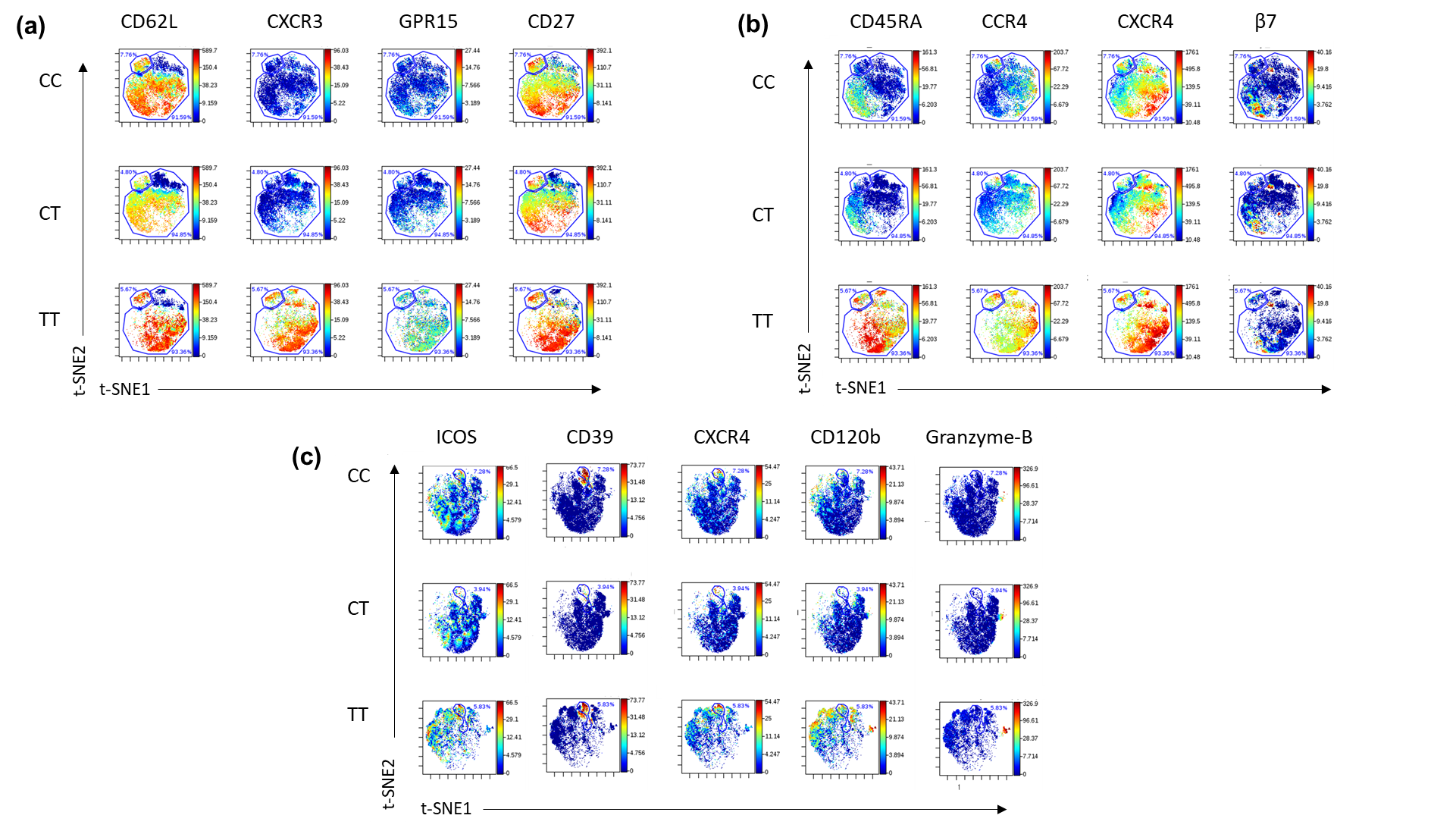
**

**Supplemental Figure 4. viSNE maps depicting expression levels of indicated markers used for Cytometry Time Of Flight analysis. a:** Expression of CD62L, CXCR3, GPR15 and CD27. Top left gate depicts regulatory T cells (T_reg_), bottom gate depicts CD4+ effector T cells (T_eff_). **b**: Expression of CD45RA, CCR4, CXCR4 and 𝛃7. Top left gate depicts T_reg_, bottom gate depicts T_eff_. **c:** Differential expression of Treg effector molecules ICOS, CD39, CTLA-4, CD120b and Granzyme B. Top left gate depicts T_reg_. for all panels, red-blue coloring denotes highest-lowest level of expression. TT – homozygotes for minor allele, CT- heterozygotes, CC – homozygotes for major allele of rs61839660.


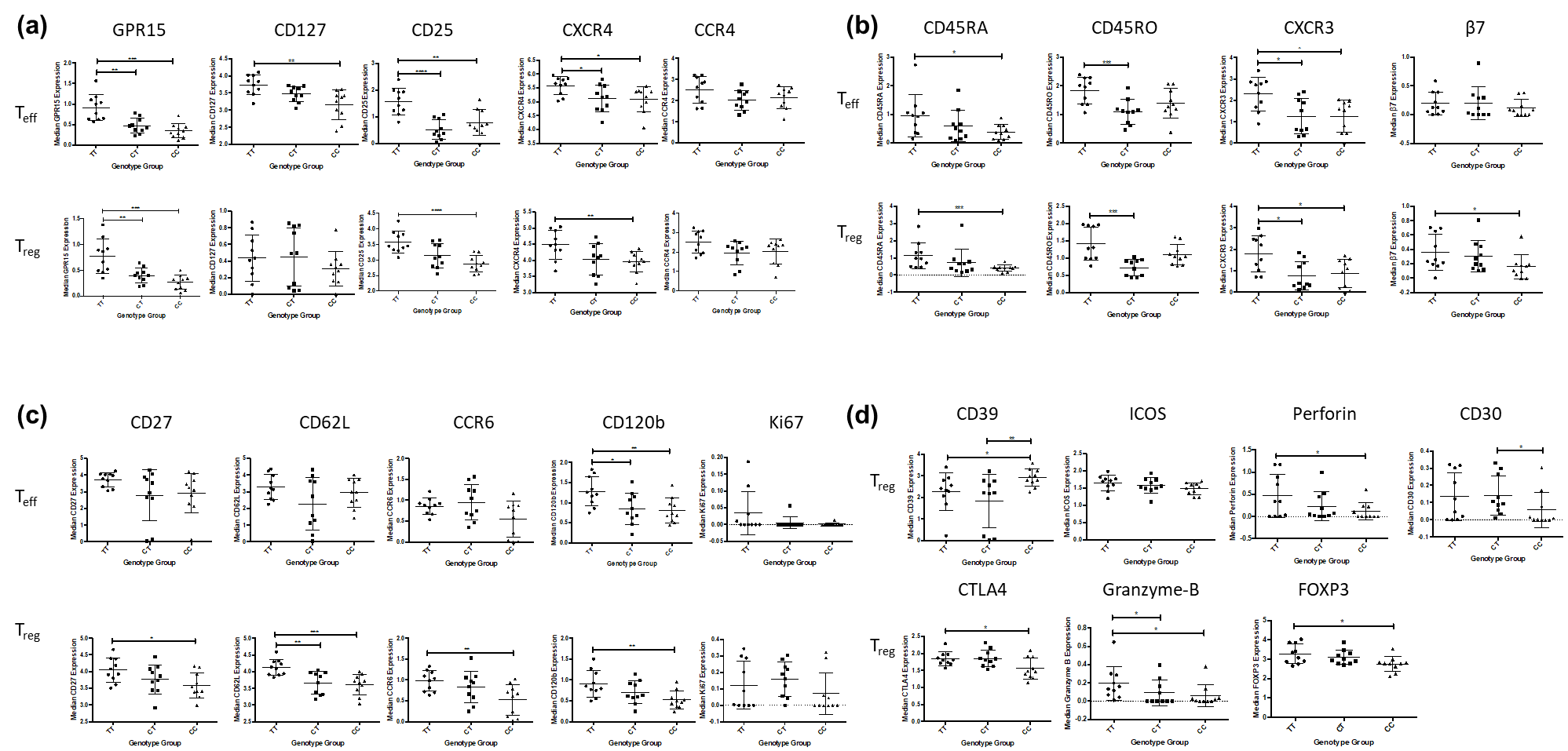


**Supplemental Figure 5. Differential expression of gut homing and effector molecules in regulatory T cells and CD4^+^ T effectors between Crohn’s Disease patient rs61839660 genotype groups. a:** Differential expression of GPR15, CD127, CD25, CXCR4 and CCR4 by regulatory T cells (T_reg_) and CD4^+^ T effectors (T_eff_) between genotypes. **b:** Differential expression of CD45RA, CD45RO, CXCR3 and 𝛃7 by T_reg_ and T_eff_ between genotypes. **c:** Differential expression of CD27, CD62L, CCR6, CD120b and Ki67 by T_reg_ and T_eff_ between patient groups. **d:** Differential expression of CD39, ICOS, Perforin, CD30, CTLA4, Granzyme-B and Foxp3 by T_regs_, between patient groups. *p<0.05, **p<0.001, ***p<0.0001. TT – homozygotes for minor allele, CT- heterozygotes, CC – homozygotes for major allele of rs61839660.
